# On Multi-Objective Based Constitutive Modelling Methodology and Numerical Validation in Small-Hole Drilling of Al6063/SiC_p_ Composites

**DOI:** 10.3390/ma11010097

**Published:** 2018-01-11

**Authors:** Junfeng Xiang, Lijing Xie, Feinong Gao, Yu Zhang, Jie Yi, Tao Wang, Siqin Pang, Xibin Wang

**Affiliations:** 1 School of Mechanical Engineering, Beijing Institute of Technology, Beijing 100081, China; xiang_junfeng@126.com (J.X); 2120170328@bit.edu.cn (F.G.); 1120110867@bit.edu.cn (Y.Z.); runless1978@sina.com (J.Y.); 18813126659@aliyun.com (S.P.); xjf2014@bit.edu.cn (X.W.); 2 National Key Laboratory of Science and Technology on Micro/Nano Fabrication, Peking University, Beijing 100871, China; mount_feng@sina.com

**Keywords:** multi-objective identification, constitutive modelling, varying weighting, measured noise, composites, drilling

## Abstract

Discrepancies in capturing material behavior of some materials, such as Particulate Reinforced Metal Matrix Composites, by using conventional ad hoc strategy make the applicability of Johnson-Cook constitutive model challenged. Despites applicable efforts, its extended formalism with more fitting parameters would increase the difficulty in identifying constitutive parameters. A weighted multi-objective strategy for identifying any constitutive formalism is developed to predict mechanical behavior in static and dynamic loading conditions equally well. These varying weighting is based on the Gaussian-distributed noise evaluation of experimentally obtained stress-strain data in quasi-static or dynamic mode. This universal method can be used to determine fast and directly whether the constitutive formalism is suitable to describe the material constitutive behavior by measuring goodness-of-fit. A quantitative comparison of different fitting strategies on identifying Al6063/SiC_p_’s material parameters is made in terms of performance evaluation including noise elimination, correlation, and reliability. Eventually, a three-dimensional (3D) FE model in small-hole drilling of Al6063/SiC_p_ composites, using multi-objective identified constitutive formalism, is developed. Comparison with the experimental observations in thrust force, torque, and chip morphology provides valid evidence on the applicability of the developed multi-objective identification strategy in identifying constitutive parameters.

## 1. Introduction

Particulate Reinforced Metal Matrix Composites (PRMMCs) present low-temp properties, high strength-to-weight ratio, good wear resistance, and low thermal expansion coefficient, but poor machinability [1,2,3]. Due to the high-cost and time-consuming experiments in R&D of PRMMC components for achieving desired machining quality, FE simulations have been employed to investigate the mechanics of cutting, optimization of cutting processes, and redesign of cutting tool [4]. To produce the realistic simulation results, it is vital to develop a proper constitutive model of MMCs with capability to capture the mechanical behavior in cutting. A key focus for the establishment and application of a proper constitutive model is the correlation of experimental data, since it should be capable of representing the material mechanical behavior in the range of strains, rates of strain, and temperature in machining [5], and so it is necessitated to derive the material constitutive models and identify the parameters from a numbers of experimental observations in views of engineering applications. The interdependence and intercoupling amongst the constitutive variables, such as deformation history, strain rate, and temperature lead to the difficulties in quantifying material constants. It is considerably questionable to improve the classical phenomenological material models only based on some available experiment data, regardless of the reliability and measurement errors [6,7,8]. These improvements to the classic constitutive model in the original scientific hypothesis were determined simply by experimental data statistics. These improved models lacked in significant difference in nature and physical significance in constitutive equation form, and so rarely adopted in finite element modelling afterwards. The solutions to the constitutive identification for a certain material may consist in the fulfillment of the applicability for classical material model [9]. This is necessitated to make scientific assumption and the reasonable identification of constitutive parameters with suitable numerical tools. 

Most engineering materials exhibit varying deformation behavior at low (quasi-static) and high (dynamic) deformation rates or temperatures. This brings great challenges to the establishment and identification of the material constitutive model that can describe the mechanical response under different loading conditions. The Johnson-Cook (J-C) constitutive model is one of the most commonly used semi-empirical phenomenological ones for describing the plastic deformation behaviors at high strain, high strain rate, and high temperate, especially suitable for the simulation of machining processes [10]. Its distinct advantages in application over other models rely on its exactness at macro-scale and simplicity since the constitute model, characterized only using a few parameters to be identified, can describe the material behavior rather well.

In the J-C constitutive equation, the flow stress σ is assumed to be a multiplicative function of equivalent plastic strain $\varepsilon_{p}$, normalized strain rate ${\dot{\varepsilon }}^{*}=\dot{\varepsilon }\;/\dot{\varepsilon_{0}}$ with $\dot{\varepsilon_{0}}$ and $\dot{\varepsilon }$ being the reference strain rate and strain rate and temperature terms. The three terms, respectively, represent respectively the elasto-plastic, viscosity, and thermal softening effects of the material plastic behavior, as described by Johnson and Cook [11].

(1) $$\sigma =\left(A+B\varepsilon_{p}^{n}\right)\;\left[1+C\ln\left({\dot{\varepsilon }}^{*}\right)\right]\left[1-{\left(T^{*}\right)}^{m}\right]$$

The material constants (A,B,C,n,m) are identified by fitting stress-strain data from quasi-static compression and split-Hokinson pressure bar (SHPB) experiments. $T^{*}$ is a normalizing temperature term expressed by

(2) $$T^{*}=\{\begin{array}{c} 0;T<T_{\mathrm{room}}\;\;\;\;\;\;\;\;\;\;\;\;\\ \left(T-T_{\mathrm{room}}\right)/\left(T_{\mathrm{melt}}-T_{\mathrm{room}}\right)\\ 1;T>T_{\mathrm{melt}}\;\;\;\;\;\;\;\;\;\;\;\;\;\;\; \end{array};T_{\mathrm{room}}\leq T\leq T_{\mathrm{melt}}$$

where T is the material temperature, $T_{\mathrm{melt}}$ the melting point, and $T_{\mathrm{room}}$ the room or reference temperature. One of the formalism characteristics for J-C model in Equation (1) lies in identifying the decoupling relationship between strain rate and deformation temperature. 

In determining the empirical constants in J-C constitutive formalism, different strategies can be found in published literatures [12,13,14,15]. Amongst them, one strategy widely used in identifying material model for metals and alloys is an ad hoc strategy, with the following steps [15].

⇀ Identify and fix the initial yield stress A from the quasi-static test at the reference strain rate $\dot{\varepsilon_{0}}$ and room temperature $T_{\mathrm{room}}$;
⇀ Fit the quasi-static flow stress-strain curve to determine the coefficients B and n based on linear regression in Equation (3) transformed logarithmically through the elasto-plastic term in Equation (1):
  (3) $$\ln\left(\sigma -A\right)=\ln B+\mathrm{nln}\varepsilon_{p}$$
⇀ Identify the strain rate hardening coefficient C by fitting linearly the data of $\sigma /\left(A+B\varepsilon_{p}^{n}\right)$ against $\ln\left({\dot{\varepsilon }}^{*}\right)$ under the same strain at room/reference temperature for SHPB data from:
  (4) $$\sigma /\left(A+B\varepsilon_{p}^{n}\right)-1=C\ln\left({\dot{\varepsilon }}^{*}\right)$$
⇀ Obtain a set of C values under the different strains according to the aforementioned strategy for solving C, and average it to find a final evaluation of C;
⇀ Identify a slope value for m through the linear regression of $\ln\left(1-\sigma /\left\{\left(A+B\varepsilon_{p}^{n}\right)\;\left[1+C\ln\left({\dot{\varepsilon }}^{*}\right)\right]\right\}\right)$ against $\ln\left(T^{*}\right)$ under the same strain or rate of strain;
  (5) $$\ln\left(1-\sigma /\left\{\left(A+B\varepsilon_{p}^{n}\right)\;\left[1+C\ln\left({\dot{\varepsilon }}^{*}\right)\right]\right\}\right)=mln\left(T^{*}\right)$$
⇀ Average a set of m values calculated under different strains or rates of strain, and find an estimation of m.

One difficulty in determining the material model, however, lies in the identification of initial yield stress since in some cases the yield point of most materials is not explicitly identified. This difficulty brings a challenge to the determination of yield point, particularly for metal matrix composites [16,17]. So, here it is more appropriate to think of the coefficient A as a variable to be optimized. Furthermore, during the process of identification using the proposed methodology in Ref. [15], another difficulty in calculating strain rate-hardening coefficient C is that at different strain rates the averaged C could not adapt to each other across the strain range [18], so it is infeasible to find a suitable average value with small variance. This may be partially attributed to the randomness from the test data, or the application and exactness of material models in different material types, time, and length scales. The identifying in thermal-softening parameter m employs the same solving procedure as determining the coefficient C. To accomplish this determination using ad hoc strategy, the experimental data at different temperatures, but identical strains rates are adopted. Again, the same difficulty may come into being: the coefficient being of different orders of magnitude or large variance arrived at from different strains or strain rates data. The extended formalism of J-C constitutive model with more fitting parameters, despite applicable efforts, would increase the difficulty in identifying constitutive parameters. The major difficulties in widespread application of constitutive model for industrial simulation consist in a larger number of mechanical tests for classical identification methodology of constitutive model, and the ensuing hard identification job of material parameters involving in constitutive characterization. Additionally, large fluctuation in the stress-strain curves, especially at high strain-rate loading, may influence the reliability and accuracy of fitting constitutive model. Thus, based on experimental data from quasi-static and dynamic tests, this work is focused on the identification strategy of J-C constitutive model parameters, and provides evidence on the applicability of the developed multi-objective optimization strategy in identifying model parameters. The feasibility of constitutive model obtained using weighted multi-objective strategy is verified through the comparison of experimental and finite element results of cutting forces and chip morphologies.

## 2. Experimental Materials, Test Procedure and Results

### 2.1. Test Procedure

**Figure 1:**
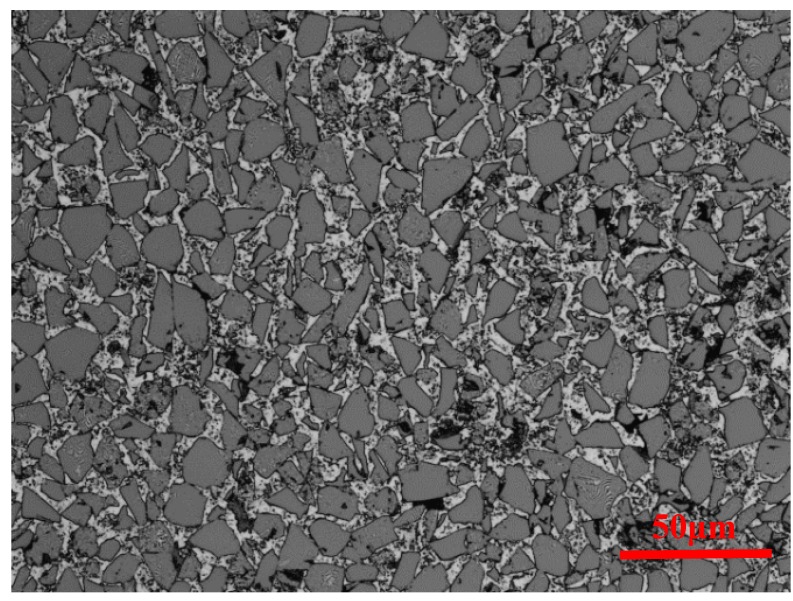
Micrograph of Al6063/SiC_p_/65p composites.

Al6063 matrix composites reinforced with 65% volume fraction of SiC particulate (Al6063/SiC_p_/65p) are investigated to focus on the mechanical responses in quasi-static compression and SHPB tests. Figure 1 shows the microstructure of Al6063/SiC_p_/65p composites, with uniformly distributed SiC particles in the absence of obvious particulate clustering and preferred orientation. At low strain rates, the temperature rise caused by plastic strain at room temperature is considered to be negligible, while at high strain rates, there exists coupling between strain rate and deformation temperature since a great amount of heat is not timely transferred. To deduce the uncoupled relationship between temperature and strain rate, the isothermal mechanical behavior at elevated temperature is to be considered in the dynamic tests at high strain rates [19].

**Figure 2:**
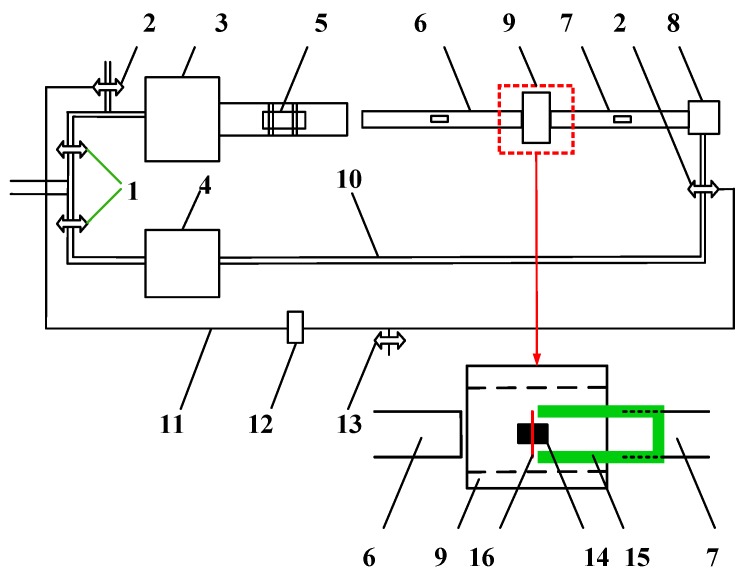
Set-up of high temperature split-Hokinson pressure bar (SHPB) equipped with a synchronically assembled heating system: 1—inlet valve, 2—magnetic outlet valve, 3 and 4—air chamber, 5—strike, 6—incident bar, 7—transmission bar, 8—plunger, 9—heating unit, 10—air pipe, 11—electric wire, 12—time rely, 13—switch, 14—sample, 15—sleeve, 16—thermocouple.

The quasi-static compression tests were carried out at a universal compression machine CMT4305 (MTS Systems Corporation, Eden Prairie, MN, USA). The specimens were machined into the cylinders of Φ6 mm × 9 mm in size. During quasi-static compression tests, the deformation rates were maintained at 10^−4^, 10^−2^, and 10^0^/s, respectively. The dynamic compression tests under high strain rates were conducted on cylinder specimens of Φ2 mm × 2 mm in size by means of SHPB. Both the quasi-static and dynamic compression tests are repeated at least three times. The SHPB testing setup at high temperatures was provided by the School of Aeronautics at Northwestern Polytechnical University (Xi'an, China). The detailed descriptions of high-temperature SHPB system and its workflow have been given in [20]. In SHPB tests, the stress-strain measure at the strain rates ranging from 10^2^ to 10^4^ was performed at room temperature. Also, the mechanical responses at loading temperatures of 300 °C and 500 °C at high strain rates were taken into account. For the high-temp SHPB tests, the specimens were first preheated to the desired test temperatures through the heating unit shown in Figure 2, and then tested in accordance with the conventional SHPB procedure [21].

### 2.2. Test Results

**Figure 3:**
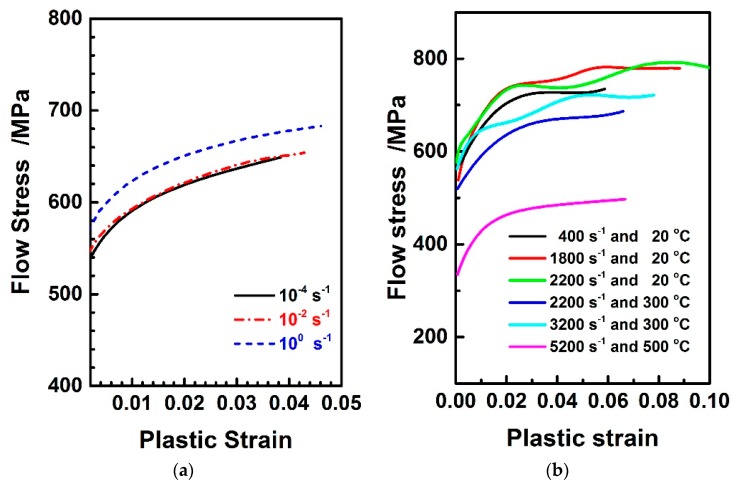
Stress-strain curves of Al6063/SiC_p_/65p composites: (**a**) Quasi-static mode; (**b**) Dynamic mode.

Figure 3a,b illustrates the stress-strain curves of Al6063/SiC_p_/65p composites in quasi-static and dynamic SHPB compression tests, respectively. It is to note that in quasi-static mode, Al6063/SiC_p_/65p composites show rate dependence to stress response, which differs from pure Al6063 alloy with no rate-dependent mechanical behavior in quasi-static compression [22]. This is mainly due to the strengthening effect caused by the impedance of the Al6063 matrix dislocation motion by SiC particulates and the transition of load bearing role from Al6063 matrix to SiC reinforcement in high volume fraction SiC reinforced composites [23]. The strain-rate hardening effect for the deformation rates ranging from 10^−4^ to 10^−2^ is not significant, so the reference strain rate in fitting constitutive model for Al6063/SiC_p_/65p composites is identified as 10^−2^. As illustrated in Figure 3b dynamic mode, the mechanical behavior of Al6063/SiC_p_/65p composites is shown to be of significant rate- and temperature-dependence. The higher the deformation rate is, the greater the data fluctuation of the measured stress-strain curves is; the higher the deformation temperature is, the less the fluctuation is. Therefore, the measurement noise at high strain rates and low temperatures become more considerable in dynamic mode, which influences the reliability and accuracy of fitted data in constitutive identification.

## 3. Parametric Identification

### 3.1. Multi-Objective Strategy Considering Weighted Measurement Errors

The J-C model in Equation (1) involves a set of five parameters (P=(A,B,C,n, m)) to be determined by matching numerous sets of measured data at quasi-static and dynamic tests. In this paper, the above parameters set identification can be turned into minimising the weighted summation of squared of aggregate errors or residuals function between the parameterized constitutive equation as a function of three independent variables ($X=(\varepsilon_{p},\dot{\varepsilon },T)$) and the sets of experimental data. That is to solve nonlinear least squares problems over all the data points. Here, a Chi-squared error criterion is used as a measure of parameter optimization.

(6) $$\chi^{2}\left(P\right)=\overset{N}{\underset{i=1}{\sum }}\omega_{i}{\left(\sigma_{i}^{\mathrm{Exp}}\left(X\right)-\sigma_{i}^{\mathrm{Model}}\left(X,P\right)\right)}^{2}$$

(7) $$\chi^{2}\left(P\right)={\left[\sigma^{Exp}-\sigma^{Model}\left(P\right)\right]}^{T}W\left[\sigma^{Exp}-\sigma^{Model}\left(P\right)\right]$$

where $\sigma^{Exp}$ is the experimental observation data of flow stress, $\sigma^{Model}$ is the constitutive model function of an independent variable vector X and five material parameters vector P to be identified (The relative error of A against the offset yield point *σ*(*ε*_p_ = 0.002) is within a range of 10%, C and n are in the range of 0–1, B and m are non-negative), N is the total number of experimental data points, and W is the weighting matrix with the diagonal weighting factor $\omega_{i}$ corresponding to the observation point i. The weighting matrix should be set to the measure errors of any observations, rather than simply to uniform weighting factor that is preferred in [16]. This is due to the fact that the measure noise under different combination of strain rates and temperature differs considerably in the total observations.

Since J-C material model does take into account different mechanical responses under the quasi-static and dynamic loadings, the Chi-square error can be subdivided into the quasi-static term $\chi_{\mathrm{quas}-\mathrm{static}}^{2}$ and dynamic term $\chi_{\mathrm{dynamic}}^{2}$ as

(8) $$\chi_{\mathrm{quas}-\mathrm{static}}^{2}\left(P_{\mathrm{Static}}\right)=\overset{N_{s}}{\underset{i=1}{\sum }}\omega_{i}{\left(\sigma_{i}^{\mathrm{Exp}}\left(X\right)-{\left(A+B\varepsilon_{p}^{n}\right)}_{i}\right)}^{2}$$

(9) $$\chi_{\mathrm{quas}-\mathrm{static}}^{2}\left(P_{\mathrm{Static}}\right)={\left[\sigma_{Static}^{Exp}\left(X\right)-\sigma_{Static}^{Model}\left(X,P_{Static}\right)\right]}^{T}W_{N_{s}}^{Static}\left[\sigma_{Static}^{Exp}\left(X\right)-\sigma_{Static}^{Model}\left(X,P_{Static}\right)\right]$$

(10) $$\chi_{\mathrm{dynamic}}^{2}\left(P\right)=\overset{N_{d}}{\underset{i=1}{\sum }}\omega_{i}{\left(\sigma_{i}^{\mathrm{Exp}}\left(X\right)-\sigma_{i}^{\mathrm{Model}}\left(X,P\right)\right)}^{2}$$

(11) $$\chi_{\mathrm{dynamic}}^{2}\left(P\right)={\left[\sigma_{Dynamic}^{Exp}\left(X\right)-\sigma_{Dynamic}^{Model}\left(X,P\right)\right]}^{T}W_{N_{d}}^{Dynamic}\left[\sigma_{Dynamic}^{Exp}\left(X\right)-\sigma_{Dynamic}^{Model}\left(X,P\right)\right]$$

where $\sigma_{Static}^{Exp}$ and $\sigma_{Dynamic}^{Exp}$ are the observation values of quasi-static and dynamic experiments, respectively; $\sigma_{Static}^{Model}$ is an elasto-plastic quasi-static material model with power-law form of $A+B\varepsilon_{p}^{n}$ and a parameter set $P_{\mathrm{Static}}=\left(A,B,n\right)$; $\sigma_{Dynamic}^{Model}$ is the same dynamic constitutive form as J-C material model in Equation (1).

Thus, a seemingly bi-objective nonlinear least squares equation involving the quasi-static and dynamic terms. According to Equations (9) and (11), an overall objective function is represented as additive relationship with different weighting factors by Equation (12).

(12) $$\chi^{2}\left(P\right)=\chi_{\mathrm{quas}-\mathrm{static}}^{2}\left(P_{Static}\right)+\chi_{\mathrm{dynamic}}^{2}\left(P\right)$$

The fluctuation in some flow stress curves corresponding to high strain-rate loading conditions observed in Figure 3b influences the reliability and accuracy of fitting constitutive model, and the same phenomena have been reported in some references on particulate reinforced metal matrix composites [24,25,26]. The major reason should be because bad signal-to-noise ratios give rise to noisy experimental measurement, such that the experimental data at high rates are difficult to represent the material behavior accurately [26]. Through residuals analysis, it is found that the measure errors of experimental points under each loading condition are almost of the same order of magnitude, but not under other conditions. Additionally, the measure errors in each condition are shown to behave as a normal distribution. These facts hold essential implication that the measure errors under each combined condition of deformation rates and forming temperatures can be assumed to be identical, and so the experimental deviation between measured data and “true” value are treated as random measured noise that satisfies Gaussian distribution $N\left(0,\theta_{\dot{\varepsilon },T}^{2}\right)$. The distribution of measured noise is of varying standard deviation $\theta_{\dot{\varepsilon },T}$ with strain rates and temperatures. The relationship between the experimental observations $\sigma^{Exp}$ and fitted model data $\sigma^{Model}$ is satisfied, as follows.

(13) $$\sigma^{Exp}=\sigma^{Model}\left(P\right)+N\left(0,\theta_{\dot{\varepsilon },T}^{2}\right)$$

Since the measure error covariance $\theta_{\dot{\varepsilon },T}^{2}$ in each condition before optimization analysis are unknown, the covariance analysis of measured error is to be performed, under the assumption of identical measure error in each condition.

(14) $$\theta_{\dot{\varepsilon },T}^{2}=\frac{1}{N_{i}^{\mathrm{Pnt}}-M_{i}^{\mathrm{Para}}+1}\sum_{j}^{N_{i}^{\mathrm{Pnt}}}{\left(\sigma_{j}^{\mathrm{Exp}}\left(X\right)-\sigma_{j}^{\mathrm{Model}}\left(X,P\right)\right)}^{2}$$

where i=1, 2,… corresponds to different experimental loading conditions in the deformation combination of various strain rates and temperature; $M_{i}^{\mathrm{Para}}$ is the model active parameters to be identified; $N_{i}^{\mathrm{Pnt}}$ is the number of observations for ith loadings combination. In the parameterized model equation, five observation points at least in number are employed for solving the constitutive equation, and thus the degrees of freedom (DoF) are set to $N_{i}^{\mathrm{Pnt}}-M_{i}^{\mathrm{Para}}+1$. The weighting factors under each loading condition can be individually determined, and the diagonal weighting factors $\omega_{i}$ under i loading condition in quasi-static and dynamic modes can be, respectively, expressed by

(15) $$\omega_{i}^{\mathrm{Static}}=\frac{1}{{\left(\theta_{\dot{\varepsilon },T}^{2}\right)}_{i}}=\frac{N_{i}^{\mathrm{Pnt}}-M_{i}^{\mathrm{Para}}+1}{\sum_{j}^{N_{i}^{\mathrm{Pnt}}}{\left(\sigma_{j}^{\mathrm{Exp}}\left(X\right)-\sigma_{j}^{\mathrm{Model}}\left(X,P_{\mathrm{Static}}\right)\right)}^{2}}$$

(16) $$\omega_{i}^{\mathrm{Static}}=\frac{N_{i}^{\mathrm{Pnt}}-M_{i}^{\mathrm{Para}}+1}{{\left[\sigma_{Static}^{Exp}-\sigma_{Static}^{Model}\left(P_{Static}\right)\right]}_{i}^{T}{\left[\sigma_{Static}^{Exp}-\sigma_{Static}^{Model}\left(P_{Static}\right)\right]}_{i}}$$

Similarly,

(17) $$\omega_{i}^{\mathrm{Dynamic}}=\frac{1}{{\left(\theta_{\dot{\varepsilon },T}^{2}\right)}_{i}}=\frac{N_{i}^{\mathrm{Pnt}}-M_{i}^{\mathrm{Para}}+1}{\sum_{j}^{N_{i}^{\mathrm{Pnt}}}{\left(\sigma_{j}^{\mathrm{Exp}}\left(X\right)-\sigma_{j}^{\mathrm{Model}}\left(X,P\right)\right)}^{2}}$$

(18) $$\omega_{i}^{\mathrm{Dynamic}}=\frac{N_{i}^{\mathrm{Pnt}}-M_{i}^{\mathrm{Para}}+1}{{\left[\sigma_{Dynamic}^{Exp}-\sigma_{Dynamic}^{Model}\left(P\right)\right]}_{i}^{T}{\left[\sigma_{Dynamic}^{Exp}-\sigma_{Dynamic}^{Model}\left(P\right)\right]}_{i}}$$

So, based on the definition of weighting matrix, this seemingly bi-objective optimization actually in practice is a multi-objective minimization problem relying on the number of loading condition combinations. Thus, the following aim is to minimize the bi-objective reduced Chi-squared error summation for the flow stress data experimentally determined from different loading conditions. An updated Levenberg-Marquardt formula based on the gradient scaling is employed in solving Equation (12) [27].

(19) $$\left[J^{T}WJ+\lambda_{\mathrm{Norm}}\mathrm{diag}\left(J^{T}WJ\right)\right]=J^{T}W\left[\sigma^{Exp}-\sigma^{Model}\left(P\right)\right]$$

where $\lambda_{\mathrm{Norm}}$ is a normalized damping factor to the leading diagonal element in $J^{T}WJ+\lambda I$ from standard Levenberg-Marquardt formula. The component $J_{ij}$ at the ith observation with respect to the jth parameter in the J matrix for J-C model can be defined as

(20) $$\left\{ \begin{array}{c} J_{i1}=\left[1+C\ln\left({\dot{\varepsilon }}^{*}\right)\right]\left[1-{\left(T^{*}\right)}^{m}\right]\\ J_{i2}=\varepsilon_{p}^{n}\left[1+C\ln\left({\dot{\varepsilon }}^{*}\right)\right]\left[1-{\left(T^{*}\right)}^{m}\right]\\ J_{i3}=B\varepsilon_{p}^{n}\ln\left(\varepsilon_{p}\right)\left[1+C\ln\left({\dot{\varepsilon }}^{*}\right)\right]\left[1-{\left(T^{*}\right)}^{m}\right]\\ J_{i4}=\ln\left({\dot{\varepsilon }}^{*}\right)\left(A+B\varepsilon_{p}^{n}\right)\;\left[1-{\left(T^{*}\right)}^{m}\right]\\ J_{i5}=-{\left(T^{*}\right)}^{m}\ln\left(T^{*}\right)\left(A+B\varepsilon_{p}^{n}\right)\;\left[1+C\ln\left({\dot{\varepsilon }}^{*}\right)\right] \end{array}\right.$$

**Figure 4:**
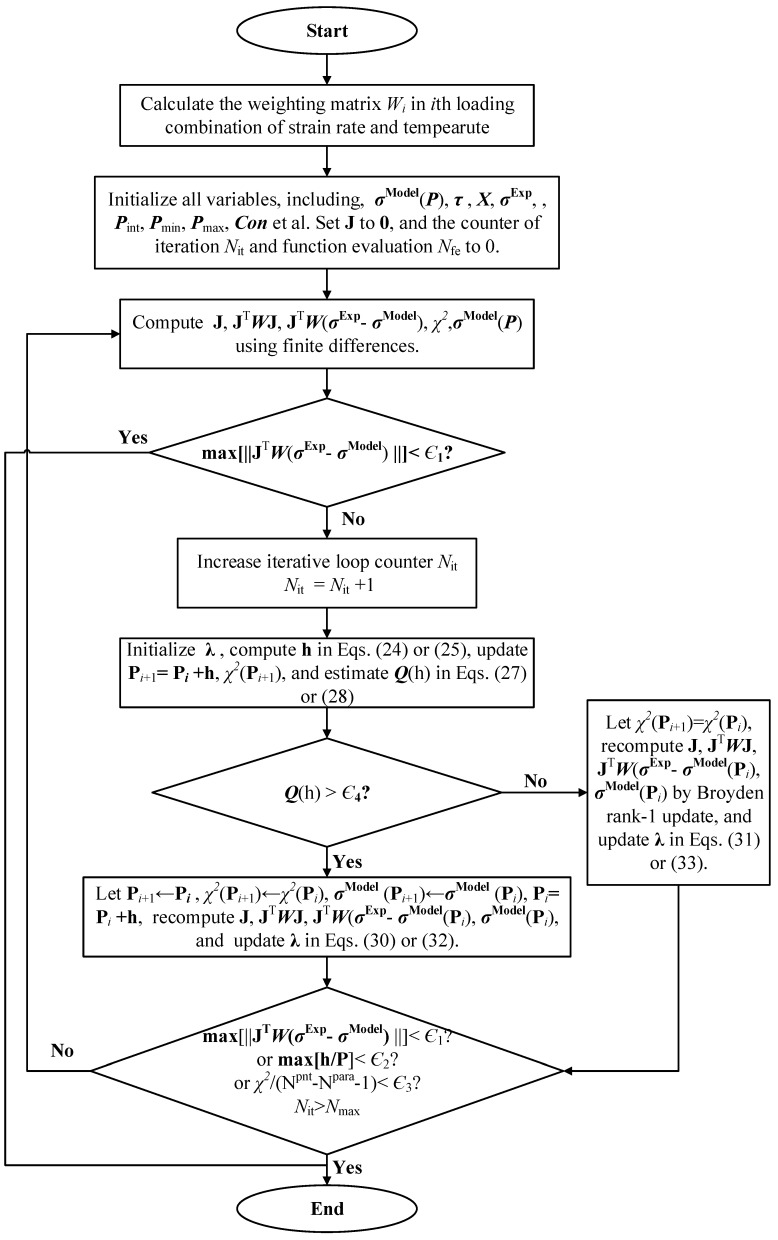
Flowchart of multi-objective optimization strategy for identifying J-C parameters.

Initialization and update of damping factor λ, Jacobian matrix J and step length h as well as convergence and error criteria are described detailedly in Appendix A. Figure 4 show the flowchart of multi-objective optimization strategy for identifying J-C parameters.

### 3.2. Identification Results and Discussion

**Figure 5:**
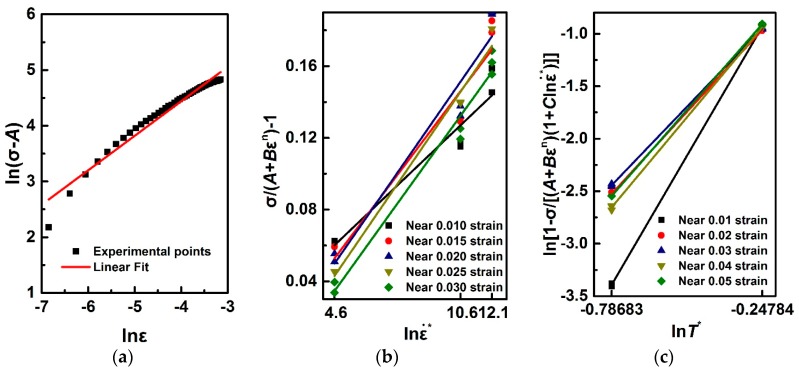
Relationship of (**a**) ln(σ−A) against lnε; (**b**) $\sigma /\left(A+B\varepsilon^{n}\right)-1$ against $\ln{\dot{\varepsilon }}^{*}$ and (**c**) $\ln\left[1-\sigma /\left(A+B\varepsilon^{n}\right)\left(1+C\ln{\dot{\varepsilon }}^{*}\right)\right]$ against $\ln T^{*}$.

Based on the relationship of ln(σ−A) against lnε in quasi-static mode, $\sigma /\left(A+B\varepsilon^{n}\right)-1$ against $\ln{\dot{\varepsilon }}^{*}$ and $\ln\left[1-\sigma /\left(A+B\varepsilon^{n}\right)\left(1+C\ln{\dot{\varepsilon }}^{*}\right)\right]$ against $\ln T^{*}$ in dynamic mode, the ad hoc strategy in Equations (3)–(5) is employed for identifying material parameters A, B, n, C, m, as shown in Figure 5. A quantitative comparison of different fitted strategies and solving algorithms for Al6063/SiC_p_/65p material model identification are summarized in Table 1. When compared to ad hoc optimization strategy, multi-objective strategy applied in J-C model optimization leads to a higher predictability with a high value of R squared (*R*^2^). This indicates that it is not always necessary to modify such a classic constitutive model under scientific hypothesis that the parameter coefficient A is a variable to be optimized rather than offset yield point. Besides, the overall fit standard error of 21.88 MPa indicates the model fitting errors are of the same order as the measured ones. That is to say, the multi-objective optimization model can be relatively capable of fitting experimental noise.

**Figure 6:**
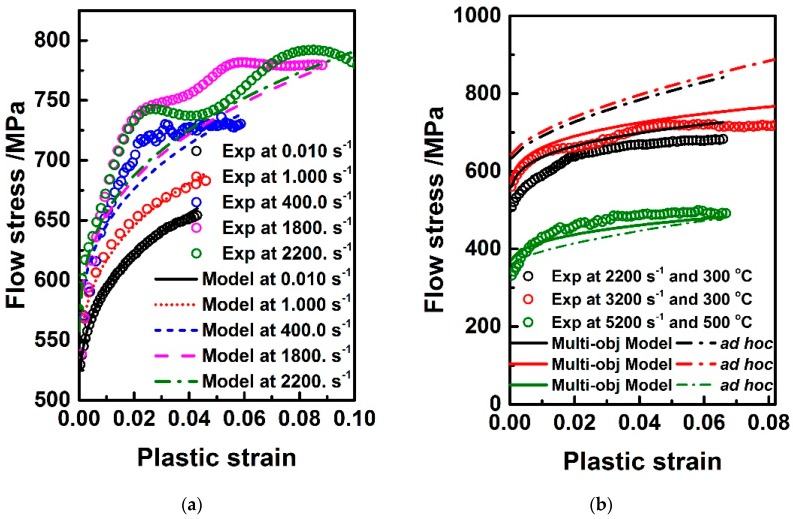
Comparison of (**a**) experimental observations and multi-objective identified model prediction; (**b**) different strategies at high temperatures and high strain rates.

Figure 6 shows the experimental observations and model prediction using J-C model parameters that were identified by multi-objective optimization strategy. It can be seen from the Figure 6a that multi-objective model prediction agrees equally well with the experiment observations in quasi-static and dynamic deformation modes. The higher visual difference between the observations and model prediction at high deformation rates such as 1800 s^−1^ and 2200 s^−1^ is mainly attributed to the experimental measurement resulting from poor signal-to-noise ratio, which easily occurs in the SHPB tests for particulate reinforced metal matrix composites. Hence, the important significance of introducing the varying weighting factors under different loading conditions into formulating multi-objective function is manifested in this aspect. When compared to the ad hoc strategy, the multi-objective optimization one enables the identified J-C model to fit the observations under high temperatures and high strain rates more accurately, in Figure 6b.

## 4. Materials and Methods

An accurate and reliable set of J-C material parameters under various combination of strain rates and temperatures could give rise to similar chip morphologies and cutting forces to experimental observations in cutting [28,29,30]. The workpiece parts in contact with the drill from the lip and axis centre tend to experience a wide range of strains, strain rates, and temperatures. So, the feasibility of constitutive model obtained using weighted multi-objective strategy is verified through the comparison of experimental and finite element results of drilling.

### 4.1. Materials

The J-C material model for Al6063/SiC_p_/65p composites is given in Table 1. For damage initiation and evolution, a combination of Johnson-Cook and shear failure criteria is used for representing ductile fracture caused by crack nucleation, growth, and voids coalescence in Al matrix and shear facture induced by local shear band in machining. A Johnson-Cook criterion for damage onset is modified for the formula put forward by Johnson [31], as

(21) $$\varepsilon_{p}^{D}\left(\eta ,{\dot{\bar{\varepsilon }}}_{p}\right)=\left(d_{1}+d_{2}\exp\left(-d_{3}\eta \right)\right)\;\left[1+d_{4}\ln\left({\dot{\varepsilon }}^{*}\right)\right]\left[1+d_{5}T^{*}\right]$$

where $\varepsilon_{p}^{D}$ is the equivalent plastic strain at the onset of damage, and $d_{1}-d_{5}$ are the material damage parameters, η is the stress triaxiality with η=p/q wherein p is the hydrostatic pressure, and $\tau_{\max}$ is the shear strength. Here the Johnson-Cook damage criterion is used to describe the ductile damage of Al6063 matrix tearing.

**Figure 7:**
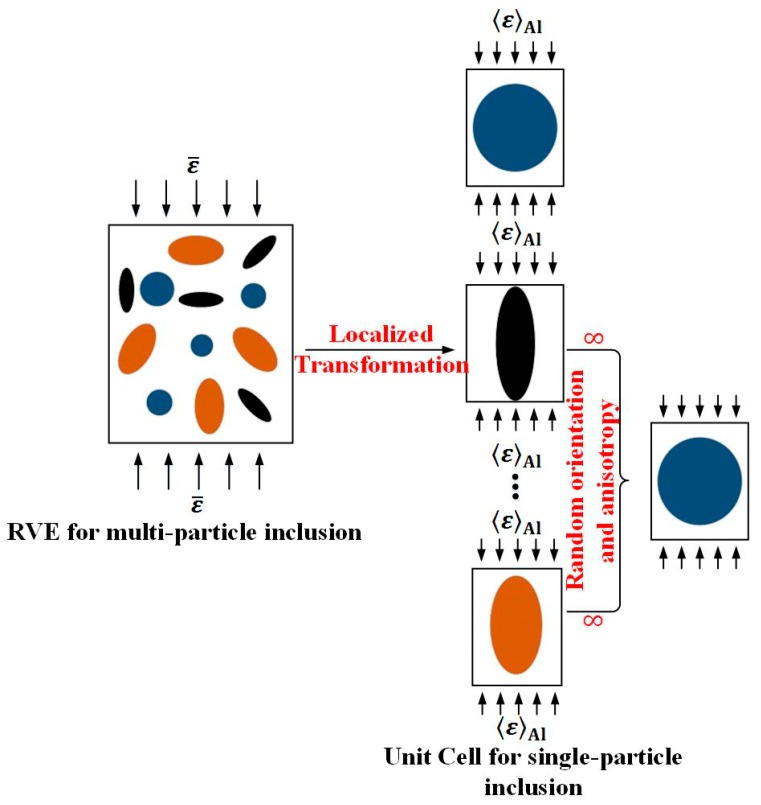
Localized relationship between representative volume element (RVE) for multi-particle inclusion and Unit Cell for single-particle inclusion.

In terms of macro- and microscopic strain-stress relationships under homogeneous strain boundary conditions shown in Figure 7, the effective strain $\bar{\varepsilon }$ of representative volume element (RVE) for two-phase constituent composites can be defined by [32].

(22) $$\bar{\varepsilon }=\left(1-V_{\mathrm{SiC}}\right){\left\langle \varepsilon \right\rangle }_{\mathrm{Al}}+V_{\mathrm{SiC}}{\left\langle \varepsilon \right\rangle }_{\mathrm{SiC}}$$

where ${\left\langle \varepsilon \right\rangle }_{\mathrm{Al}}$ and ${\left\langle \varepsilon \right\rangle }_{\mathrm{SiC}}$ is the averaged strains of Al6063 matrix and SiC phase. ⟨·⟩ denotes the volume average of physical and mechanical properties. For notational simplicity, let $\alpha_{t}=\left(1-V_{\mathrm{SiC}}\right)$.

In macro scale, due to the polycrystalline aggregates characteristic and randomly oriented distribution of SiC particle in as-cast Al6063 composites, the mechanical responses of Al6063/SiC_p_/65p composites are roughly considered macroscopically isotropic. The plastic strain part on both sides for Equation (22) should be equal, and SiC particulate is elastically deformed, and therefore the failure criterion for Al6063 matrix composites can be approximately deduced using Equations (21) and (22) as

(23) $$\varepsilon_{\mathrm{Al}-\mathrm{SiC}}^{D}\left(\eta ,{\dot{\bar{\varepsilon }}}_{p}\right)=\alpha_{t}\varepsilon_{\mathrm{Al}}^{D}\left(\eta ,{\dot{\bar{\varepsilon }}}_{p}\right)$$

(24) $$\varepsilon_{\mathrm{Al}-\mathrm{SiC}}^{D}\left(\eta ,{\dot{\bar{\varepsilon }}}_{p}\right)=\alpha_{t}\left(d_{1}^{\mathrm{Al}}+d_{2}^{\mathrm{Al}}\exp\left(-d_{3}^{\mathrm{Al}}\eta \right)\right)\left[1+d_{4}^{\mathrm{Al}}\ln\left({\dot{\varepsilon }}^{*}\right)\right]\left[1+d_{5}^{\mathrm{Al}}T^{*}\right]$$

Equation (23) is normalized to obtain the failure parameters.

(25) $$\varepsilon_{\mathrm{Al}-\mathrm{SiC}}^{D}\left(\eta ,{\dot{\bar{\varepsilon }}}_{p}\right)=\left(\alpha_{t}d_{1}^{\mathrm{Al}}+\alpha_{t}d_{2}^{\mathrm{Al}}\exp\left(-d_{3}^{\mathrm{Al}}\eta \right)\right)\;\left[1+d_{4}^{\mathrm{Al}}\ln\left({\dot{\varepsilon }}^{*}\right)\right]\left[1+d_{5}^{\mathrm{Al}}T^{*}\right]$$

The Johnson-Cook damage parameters $d_{1}^{\mathrm{Al}}-d_{5}^{\mathrm{Al}}$ for 6063 aluminum alloy were given in [33]. The Johnson-Cook failure parameters for Al6063/SiC_p_/65p composites are deduced by Equation (25), as shown in Table 2.

The shear criterion is a phenomenological one for describing shear band localization, with the following form [34]

(26) $$\varepsilon_{p}^{S}\left(\theta_{s},{\dot{\bar{\varepsilon }}}_{p}\right)$$

and

(27) $$\theta_{s}=\left(q+k_{s}p\right)/\tau_{\max}$$

where $\varepsilon_{p}^{S}$ is the equivalent plastic strain at damage initiation, q the effective Mises stress, and $k_{s}$ material parameter.

A scalar-valued damage factor ω is a measure of damage initiation. For J-C criterion in Equation (21), have

(28) $$\omega =\int^{}\frac{d\varepsilon_{p}}{\varepsilon_{p}^{D}\left(\eta ,{\dot{\bar{\varepsilon }}}_{p}\right)}=1$$

For shear criterion in Equation (26), have

(29) $$\omega =\int^{}\frac{d\varepsilon_{p}}{\varepsilon_{p}^{S}\left(\theta_{s},{\dot{\bar{\varepsilon }}}_{p}\right)}=1$$

When the damage factor ω in Equation (28) or Equation (29) reaches 1, the onset of damage is deemed to initiate.

Provided that material damage is initiated, the stress-strain law originally based on J-C material model no longer describes the material deformation behavior accurately [35]. To avoid energy-dissipated effect arising from refining mesh, a Hillerborg’s fracture energy proposal is introduced to lower mesh dependency by employing a stress-displacement law after material damage occurs. The Hillerborg’s concept is defined through fracture energy equation $G_{f}$.

(30) $$G_{f}=\int_{\varepsilon_{p}^{d}}^{\varepsilon_{p}^{f}}L\sigma_{y}d\varepsilon_{p}=\int_{0}^{u_{p}^{f}}\sigma_{y}du_{p}$$

where the element characteristic length L is introduced to represent the effective plastic displacement $u_{p}$ after damage initiation, $\sigma_{y}$ is yield stress, $\varepsilon_{p}^{d}$ is the plastic strain at the onset of damage, $\varepsilon_{p}^{f}$ and $u_{p}^{f}$ are the plastic strain and displacement at failure, respectively.

The overall damage variable D is a comprehensive measure of all active damage criteria. The damage evolution law in a maximum sense is employed for predicting damage, which is calculated as the maximum of the individual damage variable $d_{j}$ (j = 1 or 2, corresponding to Johnson-Cook damage and shear damage).

(31) $$D=\max\left(d_{j}\right)$$

A linear softening law of flow stress with equivalent plastic displacement $u_{p}$ is assumed as

(32) $$d_{j}=\frac{L\varepsilon_{p}}{u_{p}^{f}}=\frac{u_{p}}{u_{p}^{f}}$$

and in terms of Equation (30), have

(33) $$u_{p}^{f}=\frac{2G_{f}}{\sigma_{y}}$$

After damage initiation, the effective flow stress $\bar{\sigma }$ is given as

(34) $$\bar{\sigma }=\left(1-D\right)\sigma_{J-C}$$

Therefore, when the value of D reaches 1, the material failure occurs, accompanied by element deletion.

For Al6063/SiC_p_/65p composites involving α-SiC hard phase and Al6063 matrix, the specific heat capacity can be estimated according to the rule of mixtures by Equation (35).

(35) $$C_{p}=V_{\mathrm{SiC}}C_{p}^{\mathrm{SiC}}+\left(1-V_{\mathrm{SiC}}\right)C_{p}^{\mathrm{Al}6063}$$

where $C_{p}^{\mathrm{SiC}}$ and $C_{p}^{\mathrm{Al}6063}$ are the specific heat capacity of α-SiC and Al6063. The physical and mechanical properties of Al6063/SiC_p_/65p composites for material modeling are listed in Table 3.

### 4.2. Methods

#### 4.2.1. Finite Element Modelling of Drilling

**Figure 8:**
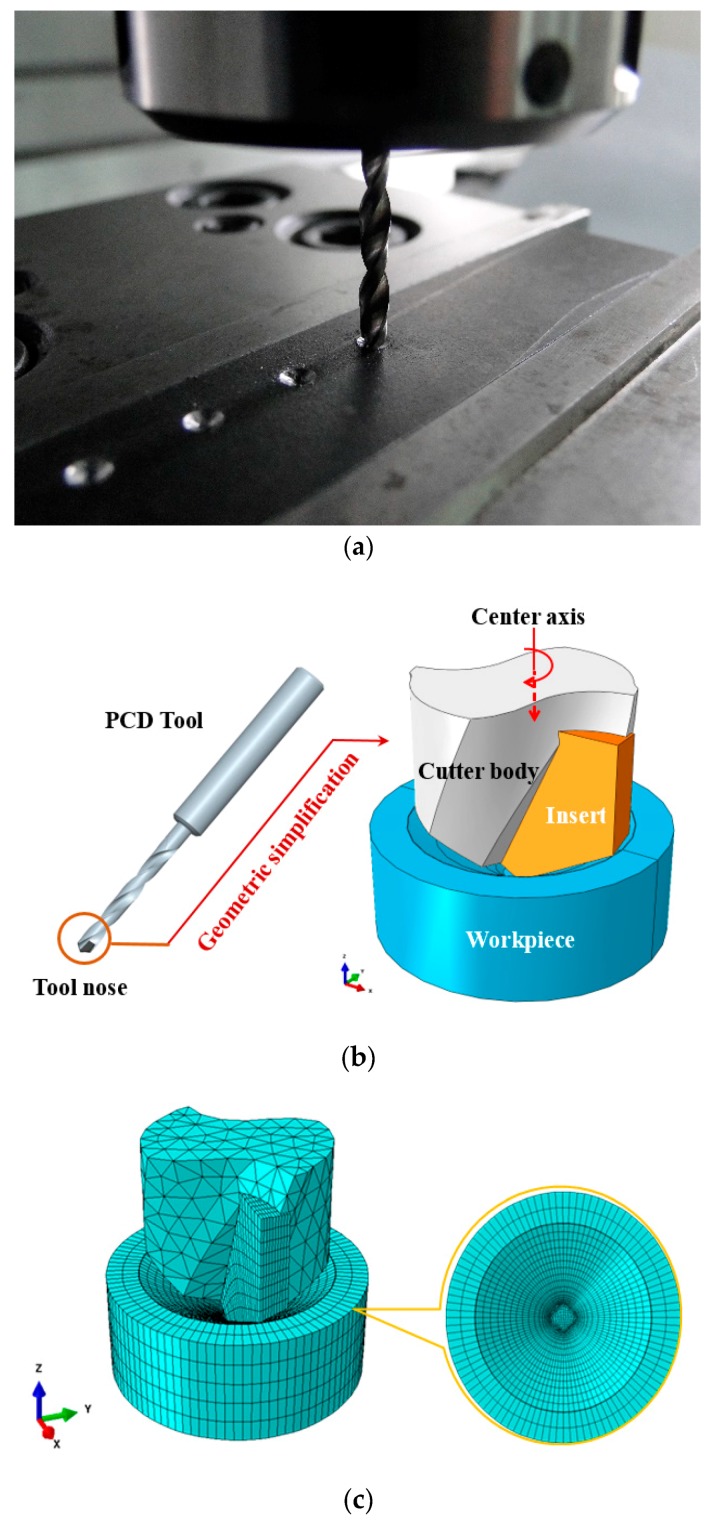
FE modelling for drilling: (**a**) Machined cone-like concave machined surface; (**b**) Geometric simplification; (**c**) Mesh generation.

A three-dimensional (3D) drilling model based on FE-based appoach has been built for simulating small-hole manufacturing process of Al6063/SiC_p_/65p composites. For workpiece modelling, the workpiece part neighbored to the tool tip are accounted for in drilling model, and a cone-like concave machined surface is prefabricated on the workpiece surface, such that stable drilling can be arrived at as soon as possible, as shown in Figure 8a. Modelling the workpiece in such a way makes it convenient to generate the structured mesh in uncut chip along the spiral cutting path so as to facilitate the formation of chip, and to enable the improvement of computational efficiency. Meanwhile, on account of the limited workpiece’s model size and the possible action of the reflected stress waves from the workpiece’s cylindrical boundary surface where more extra material exists beyond this boundary in the actual drilling infinite elements are incorporated into the boundary domain. To facilitate the implementation of infinite element modelling in which is limited to hexahedral meshes and sweep algorithm in ABAQUS, the workpiece is modelled as a cylinder with 4 mm in diameter and 1.5 mm in height. To equilibrate the computation cost and accuracy, the modeling for drill bits are only focused on the tool tip part involving the realistic cutting of the workpiece, and the sharp edges is taken into account. The PCD tool is deemed as a rigid body. The boundary conditions for the axial feed and rotation of tool are imposed on a reference point on the tool center axis, as shown in Figure 8b. The geometric simplification of drill bit and the assembly configuration of drilling model are illustrated in Figure 8b. An 8-node linear brick C3D8R with reduced integration and hourglass control is adopted for mesh division of drilling model, with the minimum mesh size of 10 μm.

It is worth noting that at least five-layer locally meshed elements along the feed direction are allocated for feed per revolution, only by such meshing strategy can the chip be formed, as shown in Figure 8c. This is mainly ascribed to material damage and element deletion involved in drilling simulation, as well as edge radius if the blunted cutting tool is to be accounted for.

The stick/slip contact model across tool-chip interface is adopted, as suggested by Zorev [36], for tool-chip contact definition. The contact formation for sticking/sliping along local tangent directions is defined as in terms of the division of slipping ($\tau \leq \tau_{\mathrm{crit}}$) and sticking regions,

(36) $$\tau =\{\begin{array}{c} \mu p,\;\;\;\;\tau \leq \tau_{\mathrm{crit}}\;\\ \tau_{\mathrm{crit}},\;\;\;\;\mathrm{Others}\; \end{array}$$

where $\tau_{\mathrm{crit}}$ is the critical shear yield stress, generally $\sqrt{3}$ times lower than the tensile yield stress. 

The cutting forces and chip morphology were obtained numerically by running FE model of drilling Al6063/SiC_p_/65p composites based on the constitutive formalism determined using multi-objective optimization strategy. The thrust force is the applied resultant force on cutting tool along the z direction.

#### 4.2.2. Experimental Set-Up and Signal Processing

**Figure 9:**
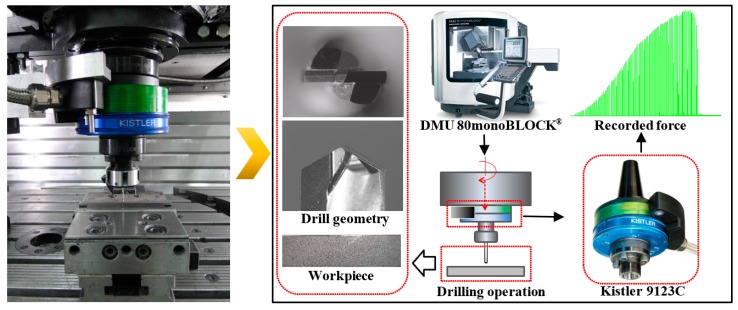
Experimental set-up.

To validate the built constitutive equation and drilling model for Al6063/SiC_p_/65p composites, the drilling experiments of Al6063/SiC_p_/65p composites were performed on a CNC machining center DMU 80 monoBLOCK (EAMTM, Brussels, Belgium), as shown in Figure 9. The measurement of the thrust forces and torque during drilling was accomplished by a rotating 4-Component Dynamometer (RCD) Kistler 9123 (Kistler Instrument China Ltd., Shanghai, China).

Due to semi-enclosed characteristics and the deformed complexity of drilling operation, the contact between the newly-formed chips by element deletion and machined surface in 3D drilling is difficult to define, so the chips flow away from the workpiece side wall, and do not impede the tool operation. This is in contradiction to the fact that in realistic drilling the newly-formed chips is inconvenient to remove, thus increasing cutting forces. To better approximate the simulation conditions, a peck drilling operation (with 0.05 mm step size) that facilitates the chip removal is employed to reduce the interference from the chips.

**Figure 10:**
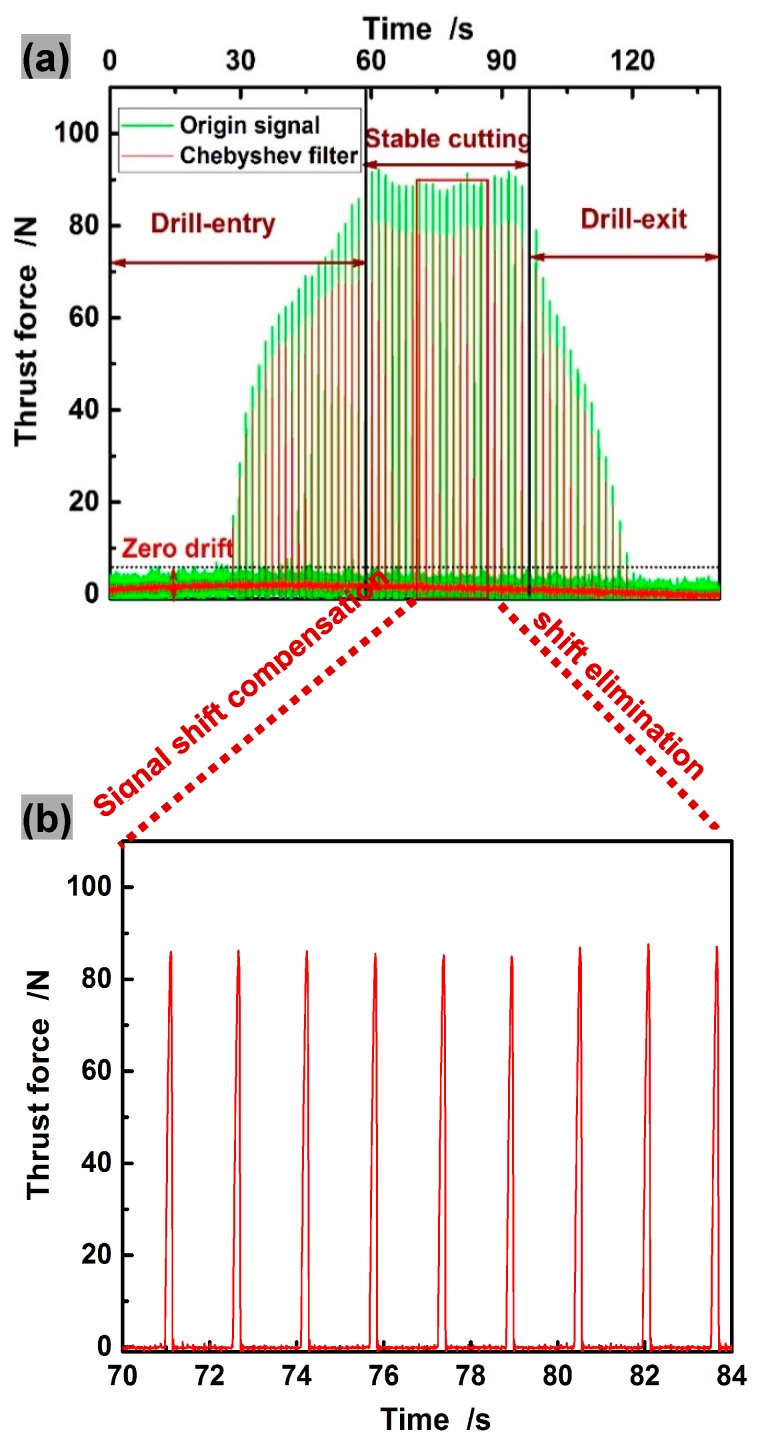
Processing and extraction of the experimentally acquired thrust force and torque signals: (**a**) Origin signal; (**b**) Processed signal.

Based on special consideration of drilling modelling, only the force signals at stable cutting are extracted for comparison. To eliminate the interference when the forcing frequencies approach natural frequencies of piezoelectric dynamometer (Figure 10a), the Type I low-pass Chebyshev filter was used for processing the experimentally acquired signals of thrust force and torque, with the cut-off frequency, as suggested by Szymon [37].

(37) $$f_{c}\approx \left(1+10\%\right)\cdot f_{\mathrm{zo}}$$

where the cut-off frequency $f_{c}$ is associated with the cutting tool kinematics represented by tooth passing frequency $f_{\mathrm{zo}}$. The already filtered signals were dealt with shift compensation for avoiding zero drift. After signal processing, the acquired thrust force signals at rotational speed of 1500 rpm and feed of 50 mm/min are shown in Figure 10b.

### 4.3. Results and Discussion

#### 4.3.1. Validation of Thrust Force and Torque

**Figure 11:**
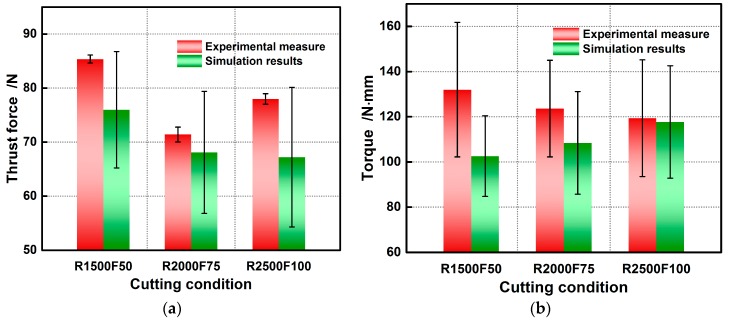
Comparison of numerically and experimentally obtained results: (**a**) Thrust force; (**b**) Torque.

Based on our drilling modelling strategy, the simulation results during stable cutting is take into account for comparison with the experimental data. When compared to the experimental observations in Figure 11, the maximum relative errors for the simulated thrust force and torque at rotational speed of 1500 rpm and feed of 50 mm/min are 11.00% and 22.27%, within the acceptable range. The errors are expected to decline as the hole diameter increases since the effects of tool geometric errors induced by sharpening and the dynamometer’s measurement error on forces disturbance are lessened. The great fluctuation of simulated force illustrated by error bar may be partly due to uneven occurrence of element failure and partly due to the tool-chip contact instability caused by the discontinuous chips formation and rake angle’s variation during cutting. The numerically and experimentally obtained thrust force and torque under other cutting conditions are compared in Figure 11. Figure 11a, which demonstrates the thrust force dependence on the drilling conditions, shows that there are the better machining conditions at rotational speed of 2000 rpm and feed of 75 mm/min. The developed FE model allows for presenting this dependence in the much more visual and usable form. The comparative results show both the simulation results of thrust force and torque are within the acceptable error ranges, and the constitutive formalism determined using multi-objective strategy can perform well in force prediction for small hole drilling.

#### 4.3.2. Validation of Chip Morphology

**Figure 12:**
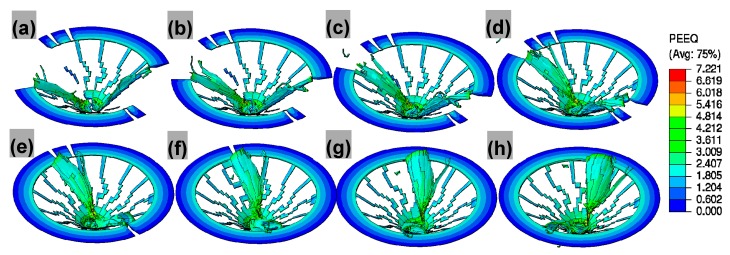
Chip formation and evolution at the rotational speed of 1500 rpm and feed of 50 mm/min: (**a**) 0.11 s; (**b**) 0.115 s; (**c**) 0.12 s; (**d**) 0.125 s; (**e**) 0.13 s; (**f**) 0.135 s; (**g**) 0.14 s; (**h**) 0.145 s.

**Figure 13:**
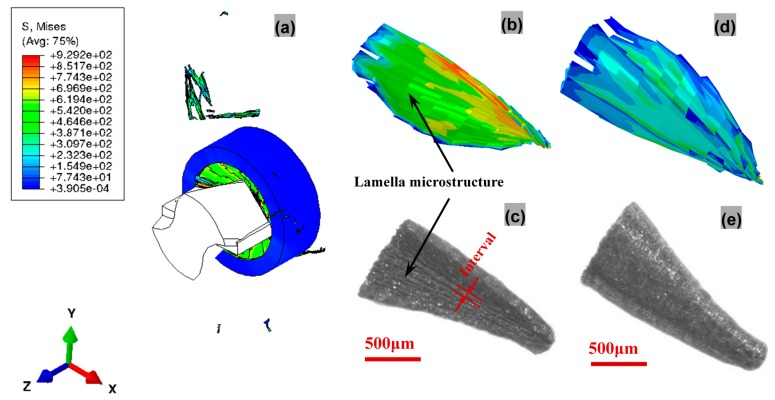
Snapshots of (**a**) FE simulation during drilling, (**b**) free surface morphologies from simulation and (**c**) experiment observation, (**d**) back surface morphologies from simulation, and (**e**) experiment observation.

The mechanical behaviors of the workpiece play a significant role in mechanics of chip formation and evolution where the workpiece material is subjected to plastic deformation and shear along the primary shear zone by dint of cutting tool [38]. The detailed chip formation and evolution mechanisms are investigated in Figure 12. Under the shearing and extruding actions from the lips and chisel edge accompanied by element failure, two approximatively centrosymmetric pieces of chip are gradually formed along with spiral downward cutting motion of drill bit. The chip is formed under the combined effects of the lips and chisel edges, and flows out along the rake face. The curling of chip is partly due to the velocity and deformation difference between the upper surface layer and the lower surface layer, and partly due to the compression from the rake face. Because the cutting lips of PCD tool is composed of two straight edges, uniform and straight morphologies of the chip root that is formed by the cutting lips are concluded to be triggered by shear failure. Less amount of chip formed by the chisel edge is mainly attributed to plastic extrusion of chisel edge with large negative rake angle, and resultant element failure is suspected of being triggered by Johnson-Cook damage criterion. Since the intersection between the cutting lips and chisel edge divides the chip into two segments, the segment formed by cutting lips is the main chips for comparison in Figure 13. The maximum equivalent plastic strain occurs right round the intersection of the margin and lip for drill bit. This is relevant to the highest cutting speed and low hydrostatic pressure here. Eventually, discontinuous chips under two damage criteria are formed, as is shown in Figure 13a. Details of the chip morphologies from simulation and experiment observation are captured in Figure 13b–e. From Figure 13b,c, the lamella morphologies from simulation and experiment observation are clearly observed in the free surface. Since the lamella morphologies result from the workpiece material’ nonuniform deformation sheared by drill bit, the interval for such microstructure is in proportion to feed per revolution during drilling. In the chip back surface, the similar morphological characteristics of the simulated chips to realistic ones again verify the validity of the drilling model and the feasibility of the developed multi-objective constitutive identification strategy with varying weighted consideration to each combination of deformation rates and temperatures.

## 5. Conclusions

In this paper, the mechanical behaviors of Al6063/SiC_p_/65p composites in quasi-static compression and SHPB tests are investigated to obtain the stress-strain responses for identifying J-C constitutive constants. Aimed at hard identification of material parameters using conventional ad hoc method, a weighted multi-objective optimization strategy for identifying J-C constitutive parameters is proposed, coupled with updated Levenberg-Marquardt implementation algorithm. When compared to ad hoc optimization strategy, multi-objective strategy applied for J-C parameters optimization leads to a higher predictability of experimental observations and better capability of fitting experimental noise. It is found that it is not always necessary to modify such a classic J-C constitutive model under scientific hypothesis that parameter the coefficient A is a variable to be optimized rather than offset yield point.

A 3D FE simulation in small hole drilling of Al6063/SiC_p_/65p composites was implemented, based on the constitutive model determined using weighted multi-objective strategy and failure criteria in a maximum sense. The maximum prediction error of no more than 11.00% for thrust forces indicates the reliability and accuracy of the proposed drilling model. It can be found from numerical and experimental results that there are the better machining conditions at rotational speed of 2000 rpm and feed of 75 mm/min. The developed FE model allows for presenting the thrust force dependence on the drilling conditions in the much more visual and usable form. Accurate prediction of thrust force is of great importance for investigating drilling induced edge defects at hole exit and entrance in PRMMCs or delamination defects in laminated composites. Hence, this proposed FE model can be used to optimize cutting processes to improve drilling performance and hole quality, based on the cutting forces effect on drilling induced defect. Accurate prediction of chip formation contributes to the disclosure of material removal mechanism. Comparative results of thrust force, torque, and chip morphology also verify the feasibility of the developed multi-objective constitutive identification strategy with varying weighted consideration to each combination of deformation rates and temperatures.

## Figures and Tables

**Table 1:** Results of J-C model identified using different fitted strategies.

Strategy	Algorithm	Parameters (*A*, *B*, n, *C*, m)	*R*^2^	OFSE
Multi-objective	Improved L-M	(453.0, 470.6, 0.2558, 0.009522, 3.954)	0.9556	21.88 MPa
ad hoc	Linear regression	(528.7, 1004.; 0.6185, 0.015400, 3.290)	0.9146	34.17 MPa

**Table 2:** Johnson-Cook failure parameters for Al6063/SiC_p_/65p composites.

$d_{1}$	$d_{2}$	$d_{3}$	$d_{4}$	$d_{5}$
0.09212	0.3647	−2.312	0.04424	2.6

**Table 3:** Physical and mechanical properties of Al6063/SiC_p_/65p composites.

Notation	Material Properties	Value
$\rho_{c}$	Density (kg/m^3^)	2960
$C_{p}$	Specific heat capacity (J/kg·°C^−1^)	750
*α*	Coefficient of thermal expansion (10^−6^ °C^−1^)	7.7
*κ*	Thermal conductivity (W/m·°C^−1^)	175
$V_{\mathrm{SiC}}$	Volume fraction of SiC (vol %)	65
E	Elastic modulus (GPa)	221
*υ*	Poisson’s ratio	0.21
$T_{\mathrm{room}}$	Room/reference temperature (°C)	20
$T_{\mathrm{melt}}$	Melting point	635
$\dot{\varepsilon_{0}}$	Reference strain rate	0.01
*η*	Inelastic heat fraction	0.9

## Appendix A. Algorithm Implementation

### A.1. Initialization and Update of Damping Factor λ

For the updated L-M algorithm in Equation (19), $\lambda_{0}$ can be specified arbitrarily in terms of fitted model. The initialization and update of $\lambda_{i}$ can follow the criteria suggested by Marquardt [28].

(A1) $$\lambda_{i+1}=\max\left[\lambda_{i}/L_{\mathrm{DN}},1.0e^{7}\right],\;Q\left(h\right)>\epsilon_{4}\;$$

(A2) $$\lambda_{i+1}=\min\left[\lambda_{i}L_{\mathrm{UP}},1.0e^{7}\right],\;\;Q\left(h\right)\leq \epsilon_{4}$$

### A.2. Update of Jacobian Matrix J

In the present paper, the Brayden rank-1 updating algorithm is employed to update Jacobian matrix, thus reducing substantially the computation cost in the absence of additional function evaluations, compared to finite different methods, especially for multi-parameter optimization problem [39].

(A3) $$J\left(P+h\right)=J\left(P\right)+hh^{T}\left[\frac{\sigma^{Model}\left(P+h\right)-\sigma^{Model}\left(P\right)}{h}-J\left(P\right)\right]/\left(h^{T}h\right)$$

However, while applying Brayden updating algorithm in the J-C model optimization, numerical unstability and divergence problems arise in the solving process. And the reason for nonconvergence, according to Broyden [32], is that in the 1st and 2$M^{\mathrm{Para}}$ iterative updating of Jacobian matrix, a bad approximation results from $\chi^{2}\left(P\right)>\chi^{2}\left(P+h\right)$. So in these iterations, the Brayden rank-1 updating algorithm is replaced by finite differences, and therefore the function evaluation is required to judge $M^{\mathrm{Para}}$ or $2M^{\mathrm{Para}}$ [40].

(A4) $$J_{ij}=\{\begin{array}{c} \frac{\sigma^{Model}\left(X_{i},{\left(P+h\right)}_{j}\right)-\sigma^{Model}\left(X_{i},P_{j}\right)}{\left\|h\right\|},h<0\;\;\\ \frac{\sigma^{Model}\left(X_{i},{\left(P+h\right)}_{j}\right)-\sigma^{Model}\left(X_{i},{\left(P-h\right)}_{j}\right)}{2\|h\|},h>0\; \end{array}$$

### A.3. Update of Step Length h

For the implementation of the algorithms above, the update of the step length h can be determined by comparing the quality of $\chi^{2}\left(P+h\right)$ against $\chi^{2}\left(P\right)$. The gain ratio Q(h), as a measure of suitable or unsuitable h, is a ratio between the actual and the expected improvement of an L-M update in the cost function [41]:

(A5) $$Q\left(h\right)=\frac{\chi^{2}\left(P\right)-\chi^{2}\left(P+h\right)}{{\left[\sigma^{Exp}-\sigma^{Model}\left(P\right)\right]}^{T}\left[\sigma^{Exp}-\sigma^{Model}\left(P\right)\right]-{\left[\sigma^{Exp}-\sigma^{Model}\left(P\right)-Jh\right]}^{T}\left[\sigma^{Exp}-\sigma^{Model}\left(P\right)-Jh\right]}$$

for Equation (19), have

(A6) $$Q{\left(h\right)}_{L-M}=\frac{\chi^{2}\left(P\right)-\chi^{2}\left(P+h\right)}{h^{T}\left(\lambda_{i}diag\left(J^{T}WJ\right)h+J^{T}W\left[\sigma^{Exp}-\sigma^{Model}\left(P\right)\right]\right)}$$

The values for $h_{i}$ is identified by judging whether $Q\left(h\right)>\epsilon_{3}$ or not, wherein $\epsilon_{3}$ is a specified threshold that determines the step acceptance in L-M algorithm.

### A.4. Convergence Criteria

The following convergence criteria are employed for iterative termination.

① Gradient criterion
  (A7) $$\max\left|J^{T}W\left[\sigma^{Exp}-\sigma^{Model}\right]\right|<\epsilon_{1}$$
② Step length increment criterion
  (A8) $$\max\left|h_{i}/P_{i}\right|<\epsilon_{2}$$
  where $\epsilon_{2}$ and $\epsilon_{3}$ are the specified thresholds for iterative termination.

### A.5. Error Criteria

The coefficient of determination, R^2^ and overall fit standard error **OFSE** are used to measure the overall quality of fit and unbiased statistical error of model predictability and reliability, respectively [42].

(A9) $$OFSE={\left[\frac{1}{N_{total}-k}\overset{N_{total}}{\underset{i=1}{\sum }}{\left(\sigma_{i}^{\mathrm{Exp}}\left(X\right)-\sigma_{i}^{\mathrm{Model}}\left(X,P\right)\right)}^{2}\right]}^{1/2}$$

where $N_{total}$ is the total experimental data point, k the number of material parameters to be fit.
